# Dr. Martin’s Report of Eleven Cases of “So-called” Malarial Hematuria

**Published:** 1891-02

**Authors:** 


### DR. MARTIN'S REPORT OF ELEVEN CASES OF "SO-CALLED" MALARIAL HEMATURIA.

---

CASE I.—Mr. B., aged twenty-six, blonde, planter. Was called in September 9, 1888. History of chronic malarial poisoning. Patient had been having chills all summer. Had been taking large doses of quinine. Had severe chill the night before. Pulse 90, temperature 102 degrees; jaundice, vomiting, urine the color of coffee grounds. Gave hypodermic of morphia to relieve vomiting; gave a purgative, and directed quinine, grains viij, every eight hours. Also, sinapism to epigastrium.

September 10.—Found all the symptoms magnified, jaundice to a deep lemon color, vomiting uncontrollable, pulse 90, temperature 101 degrees, no action from bowels, urine the color and consistency of tar. Continued treatment, also gave buchu and turpentine.

September 11.—Stupor, vomiting less troublesome, but a hiccough, which was controlled by nothing except cocaine, effect only temporary; pulse 75, temperature 99 degrees. Continued treatment, increased dose of quinia, added calomel, grains iv, every hour till six doses were taken.

September 12.—Stupor deepened, hiccough worse, urine suppressed, bowels acted unconsciously, skin a dark yellow, old gold color. Treatment continued.

September 13.—Pulse 70, temperature normal, coma.

September 14.—Pulse 100, temperature normal, coma. At 8 A. M. pulse went to 150, 180, 200, and flickered out in a few minutes.

CASE II.—C. A. D., aged thirty-five, brunette, levee contractor. Was called in October 1, 1888. History of chills for past six weeks. Patient had taken a large dose of quinine in the morning, and went on the levee works at 1 P. M.; chill at 2 P. M. and he returned to camp. On the way he urinated; urine, he said, was just like pure blood. I found him with a temperature of 104 degrees at 4 P. M., pulse 101, vomiting, eyes yellow. Gave calomel and soda ää, grains v, every two hours; quinine, grains xx, every four hours; buchu, fl., ʒj, every four hours; also, a dose of antifebrine.

October 2.—Pulse 90, temperature 99 degrees. Vomiting continues, no action from bowels, skin a dark yellow, urine suppressed, no passage since the first while coming to camp. Treatment continued. Coma supervened at 8 P. M.

October 3.—Still comatose, some small actions from bowels in the bed, urine still suppressed. Convulsive twitchings of hands and legs began; at 6 A. M. pulse 100, 6:30 pulse 150, 7:00 pulse 180. Patient died at 7:30 A. M.

CASE III.—Miss S. J., aged sixteen, brunette. Was called in February 11th, 1889. History of chills "off and on" for three months. Quinine had been given to abort a chill expected on the 11th. Severe chill that morning; saw patient at 4 P. M. Slight jaundice, no vômiting, urine clearer than in the morning, but a dark wine color. Gave a purgative, ordered eight grains of quinine every eight hours. Pulse 90, temperature 99 degrees.

February 12.—Patient worse, jaundice intense, vomiting constant, urine coffee colored. Had had an unexpected chill during the night. Severe pain and aching in lumbar region and limbs. Directed to be given: calomel, grains iv, every two hours; quinine, grains x, every four hours. Pulse 90, temperature 100 degrees.

February 13.—Pulse 95, temperature 100 degrees; still no action from bowels, jaundice deeper colored, vomiting more uncontrollable, urine scanty and more viscid. Continued same treatment; added bicarbonate of potash and buchu. Also had been trying many things to control the vomiting; nothing had any effect except hypodermic of morphia; its effect was brief and its use contra-indicated. Found cold water more useful; kept wet cloths on face and neck after a dose of medicine was given. She was thoroughly cinchonized.

February 14.—Pulse 100, temperature 99 degrees. Symptoms all more intense. A few small actions from bowels during previous night; urine almost suppressed, only a drachm or so obtained by using a catheter; this was thick, dark and grumous.

Applied a blister over liver, turpentine stupes over kidneys. Continued other treatment. Patient much averse to taking quinine; says it is killing her. Patient's mother also insists on discontinuing it. It takes all of my influence and persuasive powers to show the mother that, as the disease is malarial, and as quinine is a specific for malaria, the only hope is in quinine. If a cure does not result, it is because an insufficient quantity of the drug has been absorbed.

This, in spite of the fact that cinchonism is perfect, in spite of the fact that suppression of urine has progressed with absorption of quinine, in spite of the fact that all of the old swampers, who have fought through many a case of "hermitury" with no doctor within forty miles, have told me time and again that "quinine is rank pisin to hermitury," which advice I have scorned with a smile of superior knowledge as I turned to Bemiss and others of my distant and distinguished accomplices." "What good can come out of Nazareth?"

15.—Patient passed a quiet night, in stupor, deepening into coma as day appeared.

At 9 A. M. she roused for a short time; pulse 95, temperature normal. I gave her another dose of quinine and left.

Patient left at 9:40 A. M. Nurse said that the pulse grew very rapid just before death ensued.

CASE IV.—J. D., aged eighteen, planter, blonde. I was called to see this case August 10, 1889. History: Had been having chills all summer at intervals of from three days to a week. Had been taking quinine in large doses; chill on the 8th, with bloody urine; urine had cleared up on the 9th, and more quinine was taken; chill about midnight and urine again became bloody. When I saw him on the 10th, urine had cleared to a light wine color. There was slight jaundice, also slight nausea, but no troublesome vomiting; pulse 90, temperature 99 degrees.

Shortly before this I had decided to give my next case a chance without quinine, but this was to me such a typical case of the malarial origin of the disease, the hemaglobinuria coming with each chill and leaving soon after, that I could see but one course to pursue, i. e., to prevent the next malarial paroxysm, and with quinine.

Calomel had already been given, and was acting well, so I merely gave twenty grains of quinine. I gave so much because the patient had already been taking smaller doses and had failed to prevent the paroxysms.

August 11.—Another chill at 10 P. M. of the 10th. Urine had darkened to a coffee color during the night, but was again to a wine color when I arrived, jaundice more marked, vomiting also present, but easily controlled.

Clearly another chill would be disastrous, and must be prevented. Directed to be given quinine gr. xx every four hours, also buchu and turpentine; bowels open, tongue but little coated, pulse 100, temperature 99 degrees. Chill came on at 4 P. M., very little fever. Passed urine three times more; the first was darker, the next coffee colored, the next very dark, grumous, tarry; then followed suppression.

August 12.—Stupor supervened at about 8 A. M., coma at about 10 A. M., death at noon.

Soon after this I took time to ride over to compare notes with my nearest professional neighbor, Dr. Barton, of Malone's Landing. I found him suffering from an experience as bad and uncomfortable as my own. As we could not make matters worse, we decided to drop quinine from the treatment of the next few cases, at least. We decided on the simplest plan of treatment we could think of, the one used by Dr. Williams, of —— Georgia, and we pledged ourselves to try it with the next case that either of us should have.

CASE V.—J. C., aged thirty-eight, planter, brunette. Had been a lifelong sufferer from malarial poisoning; had had hematuria three times before, but had struggled through each case after months of slow convalescence. Had now been having chills for a week. On the 15th of October, 1889, he took a No. 1 capsule full of quinine every two hours until bedtime. Severe chill at 4 A. M. of the 16th, with bloody urine and vomiting, the usual jaundice coming on during the day, and there was a suppression of urine by nightfall. I was sent for several times, but was busy fifteen miles away. In the afternoon Dr. Barton was telephoned for, and arrived about midnight. He at once instituted the treatment we had decided on: Spirits turpentine, gtt. x every four hours; Epsom salts, a half tablespoonful every four hours; arsenic (Fowler's sol.), gtt. ij, and tr. ferri chloridi gtt. v, every two hours. I arrived at 6 P. M. of the 17th. Urine still suppressed. Vomiting very hard to control, bowels constipated, coated tongue with red edges, jaundice intense. Stayed with patient all night, continued the treatment and gave each dose of medicine myself. Used ice to throat and iced champagne to control vomiting. About daylight on the 18th he passed some highly colored urine, the first in thirty-six hours. Soon afterwards passed clear straw-colored urine. Bowels also acted well and the salts was dropped to be given only as needed. Convalescence began at once and progressed "without an untoward symptom." Turpentine was given in decreasing doses and abandoned on the 19th. Salts kept up as needed. As patient began to eat regularly the iron and arsenic was given after meals in correspondingly augmented doses.

Patient left for his home in Louisiana on the 27th.

CASE VI.—Boy, aged eleven, son of C. C. H., one of a family of brunettes. Was called in on the morning of the 24th of October, 1889. Typical case, scanty, bloody urine, vomiting, jaundice; and a history of many chills and much quinine. Case of two days' standing when I first saw it. I directed to be given spts turpentine gtt. vi every four hours; Epsom salts, a teaspoonful every four hours in as little water as will dissolve; Fowler's sol., gtt. j, and tinct. iron. gtt. ij, every two hours. Nourishment as often as possible. Called again at 4 P. M., found no change but was glad to see that cold water and fanning alone were controlling the vomiting.

October 25.—Very little change in patient; passed 2½ ounces of coffee-colored urine in the twenty-four hours. Bowels had moved once. Retained some broth.

October 26.—Passed five ounces of urine in twenty-four hours, the last passage wine-colored. Bowels acting freely. Vomiting allayed and patient hungry. The pulse and temperature have been the same since first day—pulse 90, temperature 99 degrees. Dropped salts.

October 27.—Passed eight ounces urine in twenty-four hours, the last a clear straw color. Skin clearing of jaundice. The mother reported some fever during the night. Judging this to be the malarial poison again asserting itself, I prescribed salicylic acid, being afraid to return to quinia, and not trusting the arsenic alone. After this there was no more fever.

October 30.—Had been receiving reports of the patient's constant improvement each day and called to discharge the case. The jaundice had disappeared, urine clear and plentiful. All treatment had stopped, except the iron and arsenic after meals and the salicylic acid. This last I ordered stopped; from the number of doses gone, I feared it had been given too often by some mistake; this the mother denied. I also observed that the patient, otherwise so much improved, had a feeble pulse, 100 beats to the minute, so I directed the mother to keep him in bed a few days longer and to feed him well. But that same evening, the mother being busy, the boy got out of bed for a romp with the children; in the midst of this he fell over and called for his mother; she came at once and placed him on the bed, where he died in a few minutes. Whether this death was caused by too much salicylic acid, or by the exertion of play in his still weak condition, or by both, I was never able to determine. It certainly was not directly from the disease, and did not dissatisfy me with the new treatment. It had done all expected of it.

This family soon removed to a locality remote from a physician and since then the mother has treated several cases of the disease with turpentine, salts and iron, having none of the other remedies, and with success in all cases.

CASE VII.—B. J. DuB., age twenty-seven, planter, blonde. History of chills at intervals all Fall. Took about twelve grains of quinine in two doses December 19, 1889. Chill at 8 P. M., urinated out of doors and did not see the color of urine. Retired but was kept awake by nausea and vomiting; urinated in a vessel at 11 P. M., urine bloody; vomiting became more frequent; urinated again at 4 A. M., urine coffee-colored. I arrived just before dawn; daylight exposed the third cardinal symptom—intense jaundice had come on in ten hours. I instituted the treatment as before: Turpentine, salts, iron, arsenic and frequent nourishment. The schedule was often varied, on account of the vomiting. Used everything at my command to allay this, but found nothing so useful and so harmless as ice water to neck and face and constant fanning. Pulse 100, temperature 101 degrees. 6 P. M. dropped salts and gave calomel instead, on account of vomiting.

December 21.—Bowels operated well and vomiting not so constant. Urine probably a shade lighter. Pulse 100, temperature 100 degrees. At 6 P. M. urine positively a shade lighter.

December 22.—Pulse 100, temperature 99 degrees. Urine a wine color. At 4:30 P. M. passed clear straw colored urine giving Hunyadi water t. i. d. now as purgative.

December 23.—Improvement steady; pulse 85, temperature 99 degrees. Time of doses changed as food is taken more regularly. Turpentine every six hours now.

December 24.—Jaundice almost gone, patient stronger. Pulse 80, temperature 99 degrees.

December 26.—A rise in temperature to 102 degrees. Patient so much recuperated that I venture five grains of quinine. It is well borne, and I direct five grains every four hours.

January 2.—No return of fever. Reduce quinine to five grains every twelve hours. The Hunyadi water to be taken as needed. Also a chalybeate tonic. Recovery rapid and complete.

CASE VIII.—Mrs. B., aged twenty-seven, type indifferent, mother of four children, youngest aged three years. Had a fever June 6th, 1890. A horseback ride on the 7th, during which she was much jarred and jolted. On the 8th another fever, and I was sent for. Found an ordinary case of intermittent; she was also five months pregnant and an abortion seemed imminent, due probably to horseback ride. Prescribed arsenic alone for the malarial feature, rest, the recumbent posture, morphia and the bromides to tide her over the danger of miscarriage. On the 9th she aborted; no fever. Fever rose on the 10th, and I was again called. Found the fever to be clearly malarial in character and treated accordingly. Gave mild purgative and directed quinine viij grains every eight hours.

No treatment appeared necessary as to the abortion, except that of the ordinary puerperal state. As uterus was firmly contracted, and os uteri no more patulous than was to be expected, and as the nurse assured me that "everything had come at once, child, afterbirth and all," I naturally was of the same belief. I was mistaken, as we shall see.

At 9 P. M. chill, moderate fever, bloody urine, vomiting.

Jan. 11.—I arrived at 10 A. M.; found pulse 90, temperature 99 degrees, urine coffee colored, jaundice intense, vomiting not marked. The lochia were normal in odor and appearance, no pain on pressure in any part of abdomen. At once stopped the quinine and began the turpentine, arsenic and iron. There was no need of a purgative, as bowels were open and acting from that given on the 10th. Urine was clear in twelve hours; jaundice disappeared in the course of two or three days. This ends the case as far as we are now concerned, but on the 12th a high fever with delirium developed, led by the now bad odor of the lochia I found that the placenta had been retained, and I now had a case of sepsis to deal with. Removed placenta, washed out womb. Continued the intra-uterine douche t. i. d. for two weeks. Patient made a very gradual but complete recovery. There was at no time a return of the hemaglobinuria. The malarial element again asserted itself during convalescence; as patient was much less anemic I ventured quinia and found it well borne. I hope that the four distinct pathological conditions in this case will not be confounded—the malarial intermittent, the abortion, the quinine hemaglobinuria and the sepsis.

CASE IX.—John M., aged thirty-five, blonde, ditcher. Had been having chills and fever for a month. I was called September 16, 1890, during a paroxysm. Patient very thin and sallow, pulse 108, temperature 104 degrees, exhibited antifebrin; left a purgative and quinia to be taken.

September 17.—Had had another chill during the night; temperature now (10 A. M.) 100 degrees, pulse 120, coffee colored urine, jaundice, nausea, but very little vomiting. Changed treatment to turpentine, salts, iron and arsenic. Nourishment as often as possible.

September 18.—Urine clearing rapidly, patient improving.

September 19.—Urine clear, jaundice less marked, bowels open. Ordered salts to be stopped.

September 20.—A rise of fever the previous night. The fever I considered malarial, as quinine hemaglobinuria is characterized by little fever. The case was doing so well that I ventured to order quinine to be taken to prevent the fever next expected. It did prevent it, but also produced a fresh invasion of hematuric symptoms, urine became a dark wine color, jaundice more marked, vomiting worse than before. Changed at once to the turpentine, iron and arsenic; the salts was not needed. Urine clear again in ten hours. Convalescence speedy. Chalybeate tonics and arsenic given until recovery was complete.

CASE X.—G. S., colored, aged thirty. The only case of well developed hemaglobinuria I have ever seen or heard of in an apparently full blood negro. I consider the case to have been genuine "malarial hematuria." There was the usual history of chills and fever and quinine. The three symptoms *par excellence* were present. Vomiting almost incessant, jaundice intense, as shown in sclera and perspiration, urine scanty and of a dark coffee-grounds color. I saw patient first Sept. 22, 1890; the above described condition had been present for several days, patient very weak and emaciated. Pulse 100, temperature 99 degrees. Gave salts, turpentine, iron and arsenic.

September 23.—Urine clearing, bowels open, nausea and vomiting allayed, retains nourishment. Pulse 100, temperature 99 degrees. Salts discontinued.

September 25.—Urine clear, appetite good, eyes a lighter yellow, pulse 90, temperature normal. Patient wants to sit up. Discharge case with cautions as to rest and quiet. Leave a tonic of iron and arsenic.

September 30.—Heard from patient, he is doing well and sitting up.

A few days after this I received word that patient had died suddenly. I have never been able to ascertain cause or mode of death.

CASE XI.—Girl, colored, aged nine. Seems to be about three eighths negro, mulatto father and quadroon mother.

September 22 is a notable day with me. It gave me my only colored cases of this diseases—this and the one preceding, but this patient is so near white that one is not surprised as in the other case. Patient has blondish tawny hair and gray eyes. History again of chills and quinine. Pulse 120, temperature $99\frac{1}{5}$ degrees, vomiting, icterus and urine the color of port wine. Same condition had been present two days when I first saw her. Same treatment instituted as in last case—turpentine, salts, iron and arsenic.

September 23.—Urine clear, bowels open.

September 25.—Patient much improved. Recovery is speedy and perfect.

I have presented four cases treated with quinine and seven cases without it. In these four cases with quinine the mortality is 100 per cent., in the seven cases without quinine the mortality is 28 per cent. or two deaths. But, as both of these deaths seem to have resulted from some outside cause and not directly from the disease, which was greatly benefited by the treatment, the mortality without quinine is really nihil.

Of course this number of cases does not warrant a drawing of percentages, but they are sufficient to furnish food for thought. True, many cases treated with more or less quinine have recovered, but the tardy convalescences and enfeebled constitutions show too well that the recoveries were in spite of the treatment.

This disease needs a name, hematuria, hemaglobinuria, etc. are merely naming symptoms, while hemorrhagic malarial fever or even hemorrhagic fever is a misnomer, true hemorrhage being rare. A name should represent the pathological condition and, as that consists in this disease of a partial dissolution of the blood, why not call the disease Lysæmia or Lyosæmia?

